# Suberoylanilide Hydroxamic Acid Treatment Reveals Crosstalks among Proteome, Ubiquitylome and Acetylome in Non-Small Cell Lung Cancer A549 Cell Line

**DOI:** 10.1038/srep09520

**Published:** 2015-03-31

**Authors:** Quan Wu, Zhongyi Cheng, Jun Zhu, Weiqing Xu, Xiaojun Peng, Chuangbin Chen, Wenting Li, Fengsong Wang, Lejie Cao, Xingling Yi, Zhiwei Wu, Jing Li, Pingsheng Fan

**Affiliations:** 1 Central Laboratory of Medical Research Centre, Affiliated Provincial Hospital, Anhui Medical University, Hefei, 230001, China; 2 Institute for Advanced Study of Translational Medicine, Tongji University, Shanghai, 200092, China; 3 Jingjie PTM Biolab (Hangzhou) Co. Ltd, Hangzhou 310018, China; 4 School of Life science, Anhui Medical University, Hefei, 230032, China; 5 Department of Respiration, Affiliated Provincial Hospital, Anhui Medical University, Hefei, 230001, China; 6 Department of Oncology, Affiliated Provincial Hospital, Anhui Medical University, Hefei, 230001, China

## Abstract

Suberoylanilide hydroxamic acid (SAHA) is a well-known histone deacetylase (HDAC) inhibitor and has been used as practical therapy for breast cancer and non-small cell lung cancer (NSCLC). It is previously demonstrated that SAHA treatment could extensively change the profile of acetylome and proteome in cancer cells. However, little is known about the impact of SAHA on other protein modifications and the crosstalks among different modifications and proteome, hindering the deep understanding of SAHA-mediated cancer therapy. In this work, by using SILAC technique, antibody-based affinity enrichment and high-resolution LC-MS/MS analysis, we investigated quantitative proteome, acetylome and ubiquitylome as well as crosstalks among the three datasets in A549 cells toward SAHA treatment. In total, 2968 proteins, 1099 acetylation sites and 1012 ubiquitination sites were quantified in response to SAHA treatment, respectively. With the aid of intensive bioinformatics, we revealed that the proteome and ubiquitylome were negatively related upon SAHA treatment. Moreover, the impact of SAHA on acetylome resulted in 258 up-regulated and 99 down-regulated acetylation sites at the threshold of 1.5 folds. Finally, we identified 55 common sites with both acetylation and ubiquitination, among which ubiquitination level in 43 sites (78.2%) was positive related to acetylation level.

Histone deacetylases (HDACs) are well known for their important functions in chromatin remodeling, cell cycle progression, cell migration suppression and epigenetic regulation impact by turning over histone lysine acetylation in various pathophysiological conditions. Moreover, HDACs are considered as important targets for cancer therapy. Therefore, HDAC inhibitors (HDACi) were emerged as practical therapies for different cancer types^1^,^2^. Furthermore, HDACi were also found to have potential therapeutic functions in cardiac conditions, arthritis and malaria^3^. As a consequence, HDACi were drawing increasing attentions in the past decade^1^,^2^,^4^,^5^,^6^ and varieties of HDACi were investigated including suberoylanilide hydroxamic acid (SAHA), depsipeptide (Romidepsin)^7^,^8^, panobinostat (LBH589)^9^ and so on. Among them, SAHA is the most studied one and was first approved by the Food and Drug Administration (FDA) as HDACi drug for the treatment of refractory cutaneous T-cell lymphomas (CTCL)^10^. In addition, its activities against other solid tumor cancers such as non-small cell lung cancer (NSCLC)^11^,^12^,^13^,^14^, breast cancer^14^,^15^,^16^ and ovarian cancer^17^,^18^ were also confirmed.

It is previously reported that SAHA can suppress tumor cell proliferation, differentiation, and can also induce cell apoptosis and cytotoxity^1^,^4^,^6^,^19^,^20^, therefore it is well-studied to show the therapeutic effect of SAHA for single treatment or combinative treatment with other small molecule inhibitors^21^,^22^. To elucidate the effect of SAHA treatment to proteins, the expression level of transcriptome and proteome in response to SAHA induction was studies. Lee et al. observed that SAHA change microRNA expression profiles in NSCLC A549 cells and breast cancer cell lines^12^. Sardiu and coworkers established a human histone deacetylase protein interaction network toward SAHA treatment^23^. In our previous study, the impacts of SAHA on proteome and histone acetylome in NSCLC A549 cells were investigated, which demonstrated that SAHA altered the profile of the whole proteome of NSCLC cells and highly increased the expression level of histone lysine acetylation, giving its intrinsic roles of HDAC inhibitor for epigenetic regulation^13^. More recently, Xu et al. found that SAHA regulate histone acetylation, butyrylation and protein expression in neuroblastoma^24^. In their study, 28 histone lysine acetylation sites and 18 histone lysine butyrylation sites were detected, most of which were up-regulated upon SAHA treatment.

Despite the extensive reports of SAHA in cancer therapy and the critical alteration of SAHA treatment to proteome and histone acetylome, the underling mechanisms are poorly understood. Previously we found that the expression levels of the global proteome and histone lysine acetylome were both regulated by SAHA treatment, and the alteration of proteome may partially be attributed to histone lysine acetylome^13^. Moreover, we also revealed that ubiquitination, a well-known PTM, is also closely related to the change of proteome level because of its important function in protein degradation^25^ and the existing crosstalk between lysine acetylation and ubiquitination^26^. Therefore, to reveal the relationship between ubiquitination and SAHA treatment, the global proteome, ubiquitylome and acetylome in response to SAHA treatment should all be studied. In this work, we established an integrated system by the combination of SILAC labeling, affinity enrichment by antibodies and high-resolution LC-MS/MS for quantitative comparison of the proteome, ubiquitylome and acetylome of A549 cells before and after SAHA treatment (Figure 1). Moreover, the crosstalk between global proteome and ubiquitylome, ubiquitylome and acetylome are also studied, which may largely deepen our understanding of SAHA-dependent NSCLC therapy.

## Results

### Integrated strategy for quantitative proteome, ubiquitylome and acetylome

SAHA is a well-studies HDAC inhibitor (HDACi) and was considered as meaningful therapy for cancers. It is reported that SAHA could induce the changes of the whole proteome expression level and increase the histone acetylation level of human NSCLC A549 cells^12^,^13^. However, the alteration of non-histone lysine acetylome was seldom explored^27^. Moreover, our results also showed that SAHA-induced proteins closely related to protein complex of ubiquitin E3 ligase^13^, which indicate that ubiquitination may also be regulated upon SAHA treatment. Therefore, the quantitative comparison of SAHA-induced proteome, ubiquitylome and acetylome is of considerable biological significance.

**Figure 1:**
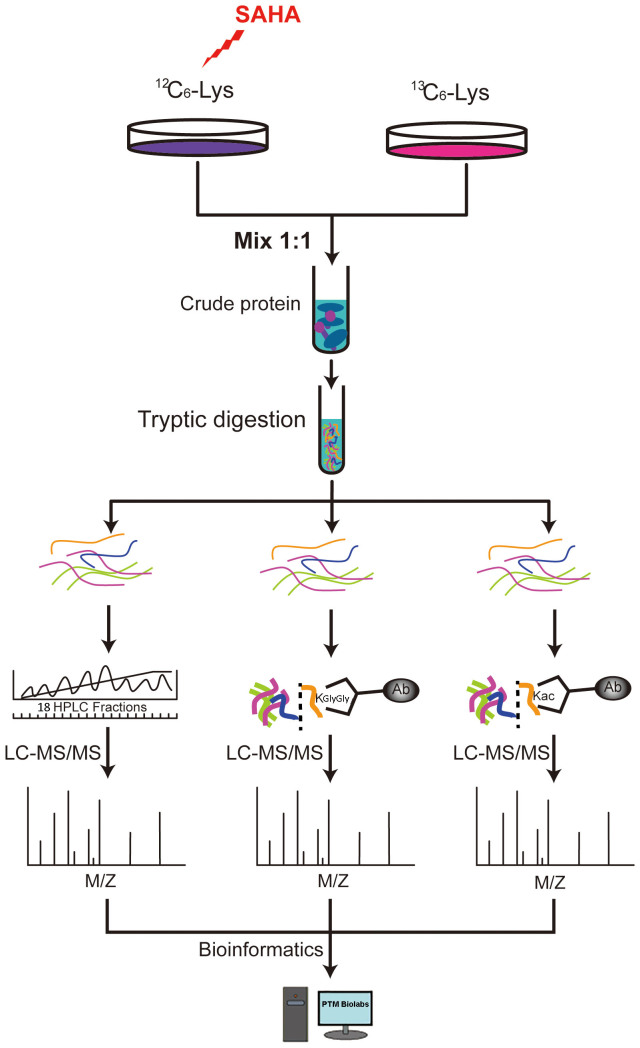
The systematic workflow for quantitative profiling of global proteome, ubiquitylome and acetylome in A549 cells upon SAHA treatment.

In this work, we combined stable isotope labeling by amino acids in cell culture (SILAC), HPLC fractionation, high specific pan-antibody enrichment, high resolution Orbitrap mass spectrometry and bioinformatic analysis for systematic quantification of proteome, lysine ubiquitylome and lysine acetylome upon SAHA treatment in A549 cells. The integrated workflow includes 6 key steps, as shown in Figure 1: (1) stable isotope labeling of A549 cells by SILAC; (2) purification and digestion of cell lysate; (3) HPLC separation of extracted proteins into fractions; (4) affinity enrichment and purification of lysine ubiquitylated and acetylated peptides; (5) separation and analysis of the enriched peptides using nano-LC-MS/MS; (6) interpretation of the collected MS data and analysis of proteome, lysine ubiquitylome and acetylome in protein functions, pathways, interaction networks and crosstalks.

### SAHA treatment changes proteome profile in A549 cells

The microRNA expression levels in different cancer cells were observed to be changed after SAHA treatment^12^. Furthermore, the alteration of the whole proteome in A549 cells in response to SAHA stimulation was also demonstrated by our previous study^13^. In this work, 4302 proteins were identified and 2968 proteins were quantified by the comparison between cells with and without SAHA treatment. Among the 2968 quantifiable proteins, 1279 were changed over 1.3 folds (598 up-regulated and 681 down-regulated) and 817 were changed over 1.5 folds (365 up-regulated and 452 down-regulated). All these data was listed in Supplementary Information Table S1.

To elucidate the functional differences of down-regulated and up-regulated proteins, the quantified proteins were analyzed for four types of enrichment-based clustering analyses: Gene Ontology (GO) enrichment-based clustering analysis, protein domain enrichment-based clustering analysis, KEGG pathway enrichment-based clustering analysis and protein complex enrichment-based clustering analysis (Figure S1A–F). As changing ratio of 1.3 and 1.5 were both used as significant threshold by lots of previous lung tumor or cancer proteomics studies^28^,^29^,^30^, we performed above clustering analyses by dividing all significantly changed proteins into four quantiles (Q1–Q4) according to L/H ratios (Q1: <0.67, Q2: 0.77–0.67, Q3: 1.3–1.5, Q4: >1.5) to see the biological functions of the proteins with large changing ratios (>1.5 or <0.67) or with relatively small changing ratios (1.3–1.5 or 0.77–0.67) upon SAHA treatment.

GO enrichment-based clustering including cellular compartment, biological process and molecular function was firstly performed (Figure S1A–C). It is observed that significant differences were occurred among different quantiles. In the cellular component category (Figure S1A), the up-regulated proteins were highly enriched in mitochondria and endoplasmic reticulum (ER), while the down-regulated proteins were enriched in nucleus, chromosome, ribosome, spliceosomal complex and transcriptional repressor complex. This is the response to SAHA treatment which suppressed the cell transcription. Moreover, proteins focused on histone methyltransferase complex and methyltransferase complex were both enriched in down-regulated proteins which demonstrated that SAHA may decrease lysine methylation in cells. In biological process (Figure S1B), the processes related to regulation of cell cycle and gene expression were enriched in proteins with low L/H ratios. The analysis of molecular functions (Figure S1C) showed that proteins involved in the binding of cofactor and coenzyme, catalytic activity were enriched toward SAHA treatment. Therefore, despite the differentially proteome pattern, the enriched nuclear, chromosome and DNA related terms accounted for relative larger proportion and these terms were extensively associated with histone lysine acetylation, suggesting the intrinsic roles of SAHA as HDAC inhibitor.

Specific domain structure is one of the major functional features in proteins. As a consequence, we next analyzed the enriched domain of those up- and down-regulated proteins induced by SAHA treatment (Figure S1D). We observed that protein domains involved in nucleic acid-binding, OB-fold, chromo and so on were enriched in down-regulated proteins, and glycoside hydrolase, EF-hand domain, linker histone H1/H5 domain H15 were enriched in up-regulated quantiles.

To identify metabolic pathways regulated by SAHA treatment, we performed a pathway enrichment-based clustering analysis by using the Kyoto Encyclopedia of Genes and Genomes (KEGG) database (Figure S1E). The results showed that lysosome, synaptic vesicle cycle, mucin type O-glycan biosynthesis, and fatty acid metabolism were the most prominent pathways enriched in quantiles with increased protein level in SAHA-treated cells, suggesting a role of SAHA in these pathways. In contrast, protein expression in the cellular pathways of DNA replication, RNA degradation, spliceosome, SNARE interactions in vesicular transport, and RNA transport was decreased in response to SAHA treatment.

By using a manually curated CORUM database, we performed enrichment analysis on protein complexes (Figure S1F). Altogether, we obtained 15 complexes with significant enrichment in Q1 and 14 complexes enriched in Q4. These complexes can be considered to be SAHA-regulated core complexes. For example, anti-HDAC2 complex and MTA1-HDAC core complex were enriched in Q1, showing the intrinsic roles of SAHA as an HDAC inhibitor. Moreover, the expression level of proteins contains spliceosome, ribosome, 60 S ribosomal subunit and 40 S ribosomal subunit were significantly down-regulated in response to SAHA treatment. These complexes are closely associated with ribosome biogenesis and protein translation, the decreased level of proteins containing these complexes suggested the non-HDACi roles of SAHA in the regulation of protein expression.

### SAHA treatment changes ubiquitylome profile in A549 cells

Ubiquitination or ubiquitylation is one of the most important post-translational modifications (PTMs) and is formed by covalent attachment of ubiquitin to its target proteins. It is well-known for its function in targeting proteins for degradation by the proteasome^25^. Moreover, it also play critical roles in cell signaling, immune system and tumor suppression^31^,^32^,^33^. In our previous study, ubiquitin E3 ligase was found to be closely related to SAHA treatment^13^, therefore the alteration of protein ubiquitylome after SAHA treatment was investigated in this work.

Proteome-wide enrichment of ubiquitination is based on its distinct feature of di-glycine remnant (K-ε-GG). In this work, we combined SILAC, immuneaffinity enrichment by a high-quality anti-K-ε-GG antibody (PTM Biolabs) and high-resolution mass spectrometry for the quantification of protein ubiquitination in A549 cells upon SAHA treatment. Altogether, we identified 1067 ubiquitination sites on 613 proteins from A549 cells, and 1012 sites from 586 proteins were quantifiable, among which 614 were changed over 1.3 folds (340 up-regulated and 274 down-regulated) and 426 were changed over 1.5 folds (234 up-regulated and 192 down-regulated). All these data was listed in Supplementary Information Table S2.

**Figure 2:**
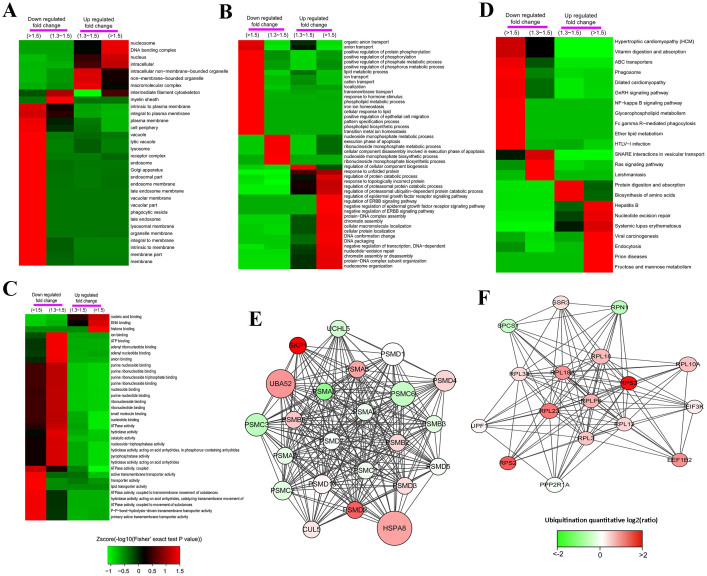
Functional enrichment-based clustering analysis for the quantified ubiquitylome. (A), cellular component analysis. (B), biological process analysis. (C), molecular function analysis. (D), KEGG pathway analysis. (E), protein-protein interaction network of ubiquitylated proteins clustered in proteasome. (F), protein-protein interaction network of ubiquitylated proteins clustered in ribosome.

For clustering analysis, all the quantified ubiquitination were also divided into four quantiles (Q1–Q4) according to L/H ratios the same as were described above. Then, the enrichment-based clustering analyses (Gene Ontology, protein domain, KEGG pathway and protein complex) were performed (Figure 2A–D and Figure S1). For the cellular component analysis (Figure 2A), we found that lots of proteins located on membrane such as membrane part, plasma membrane, endosome membrane, vacuolar membrane, lysosomal membrane, organelle membrane and so on were highly enriched in Q1 with down-regulated Kub sites. This result indicated that ubiquitination may possess important roles in cell membrane. On the contrary, the up-regulated Kub proteins were focused on nucleosome, DNA bending complex, nucleus, and intracellular. The biological process of ubiquitination was also analyzed as shown in Figure 2B. For the down-regulated Kub proteins, they were highly enriched in phosphorylation processes including positive regulation of protein phosphorylation, phosphate metabolic process and phosphorus metabolic process, which may attributed to the interaction between ubiquitination and phosphorylation^34^ and indicating that SAHA treatment could be used to suppress protein phosphorylation. Moreover, proteins with down-regulated Kub sites were also focused on ion transport processed such as anion transport, cation transport and organic anion transport, this may related to the functions of ubiquitination to cell membranes. For proteins with up-regulated Kub sites, they were enriched in ubiquitin-dependent protein catabolic process. In addition, proteins with up-regulated Kub sites were also enriched in chromatin assembly, protein-DNA complex assembly, negative regulation of transcription which suggests that ubiquitination was also related to cell cycle and transcription, the increase level of ubiquitination could slow down cell cycle and transcription. The molecular function analysis was presented in Figure 2C. It is observed that proteins with ATPase activity, hydrolase activity and transporter activity were enriched in Q1 and Q2 which is consistent with the biological process analysis results of ion transport described above. By contrast, proteins with up-regulated Kub sites in Q3 and Q4 were enriched in nucleic acid binding, DNA binding and histone binding.

For the protein domain analysis, we observed that protein domains involved in aldehyde dehydrogenase, ABC transporter and von Willebr factor were enriched in proteins with down-regulated Kub sites, and zinc finger, ubiquitin-like, histone core and histone-fold were enriched in up-regulated quantiles (Figure S2A).

The KEGG pathway analysis of the quantitatively changed proteins undergo ubiquitination showed a number of vital pathways. The pathways of ABC transporters, NF-kappa B signaling pathway and Ras signaling pathway were enriched in Q1 and Q2 with down-regulated Kub sites, while proteins with up-regulated Kub sites were enriched in Hapatitis B, systemic lupus erythematosus and so on (Figure 2D). These results showed that ubiquitination were highly associated with cell signaling and diseases^32^,^35^.

Finally, the protein complex analysis was performed and shown in Figure S2B. It was observed that proteins with up-regulated Kub sites were highly enriched in protein complexes of ubiquitin E3 ligase which was also reported in our previous study^13^. More interestingly, ubiquitination level of proteins associated with ribosome, 60S ribosomal subunit and Nop56p-associated pre-rRNA complex were also significantly up-regulated in response to SAHA treatment, which are totally opposite to that of global proteome alteration in these complexes. This phenomenon suggested that the decrease of global proteome is negatively regulated by ubiquitination.

Protein-protein interaction network of the ubiquitylated proteins was also established by using Cytoscape software (Figure S3 and Figure 2E–F). The global network among Kub proteins were shown in Figure S3. It was also observed that Kub proteins were highly enriched in proteasome and ribosome (Figure 2E–F). Proteasome is very important for ubiquitylated proteins to be degraded and we found that HSPA8 (Heat shock 70 kDa protein), a key protein in protein degradation, was ubiquitylated at multiple sites and their ubiquitination levels were all highly increased up-SAHA treatment (Figure 2E).

### Crosstalk between global proteome and ubiquitylome

Ubiquitination is well-known for its protein-degradation function by the proteasome^25^. The expression of proteins in cells may also be regulated by ubiquitination. In this work, the quantitative proteome and ubiquitylome in A549 cells toward SAHA treatment were both obtained. Therefore, the interaction between proteome and ubiquitination could be studied.

**Figure 3:**
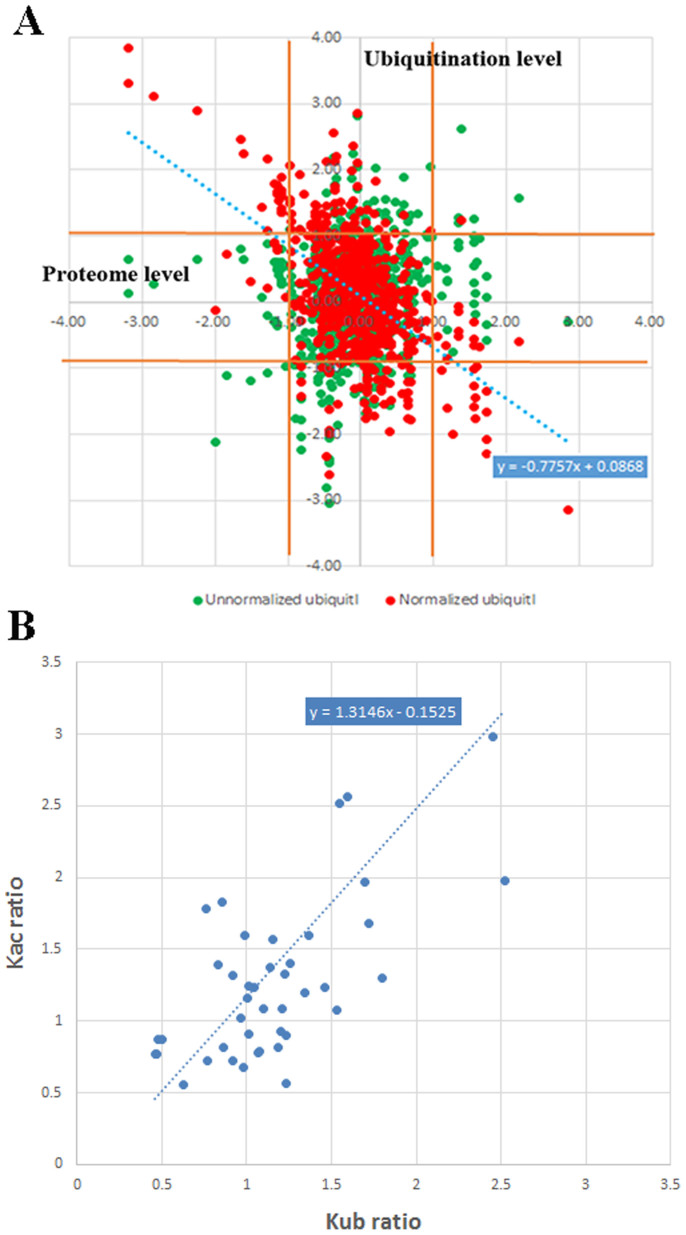
Crosstalk analysis among global proteome, ubiquitylome and acetylome. (A), crosstalk between global proteome and ubiquitylome. (B), crosstalk between ubiquitylome and acetylome.

According to the quantitative results obtained in this study, the crosstalk between the whole proteome and ubiquitylome in A549 cells was analyzed. In our data, there are 343 quantified proteins which also undergo ubiquitination, and a number of 663 Kub sites were quantified. The quantitative ratios of proteome and ubiquitylome upon SAHA treatment were compared as shown in Figure 3A and Supplementary Table S4. To be accurate, the ratio was normalized as shown in red. The pearson's correlation coefficient and the Spearman's rank correlation coefficient were calculated as −0.53 and −0.46, respectively. Therefore, the global proteome and ubiquitylome were weak negatively correlated, which imply changing pattern of proteome was opposite to that of ubiquitylome upon SAHA to some extent. This result demonstrated that the expression level of proteome was negatively regulated by ubiquitination which is consistent with the protein-degradation function of ubiquitination.

### SAHA treatment changes acetylome profile in A549 cells

SAHA is a well-known HDACi and its therapeutic functions for different cancers were extensively studies^1^,^2^,^4^,^5^,^6^. Previously we revealed that SAHA significantly increased the acetylation level on most histone Kac sites. More interestingly, it also tremendously decreased the acetylation level of some important “histone markers”^13^. In this work, the acetylation level of non-histone proteins was investigated in response to SAHA treatment by the combination of SILAC, lysine acetylation antibody enrichment and LC-MS/MS analysis.

Our study identified 1124 acetylation sites corresponding to 551 proteins from A549 cells, among which 1099 acetylation sites from 542 proteins were quantifiable (Supplementary Information Table S3). To our best knowledge, it is the most comprehensive profiling of lysine acetylation dataset upon SAHA treatment in A549 cells.

In this work, 91 Kac sites were identified from histones including various histone isoforms, among which 87 sites were quantified. The acetylation levels of most histone Kac sites (66 out of 87) were increased (>1.5 folds) and 61 Kac sites were even up-regulated over 2 folds. Only three Kac sites were down-regulated. Compared with previous results, all of the “histone markers” except one were covered by this work^13^. Moreover, H3K23ac and H4K12ac which were reported previously to be significantly decreased due to SAHA induction were also quantified as down-regulated with the changing fold of 0.33 and 0.61, respectively.

Depart from histone acetylation, we demonstrated in this study that the acetylation level of non-histone proteins was also increased by SAHA treatment. Among 1012 quantified Kac site from non-histone proteins, 296 and 258 sites were up-regulated by setting the ratio bar of 1.3 and 1.5 folds, respectively. However, there are still 178 and 99 sites down-regulated with ratio <0.77 and 0.67 fold upon SAHA treatment.

**Figure 4:**
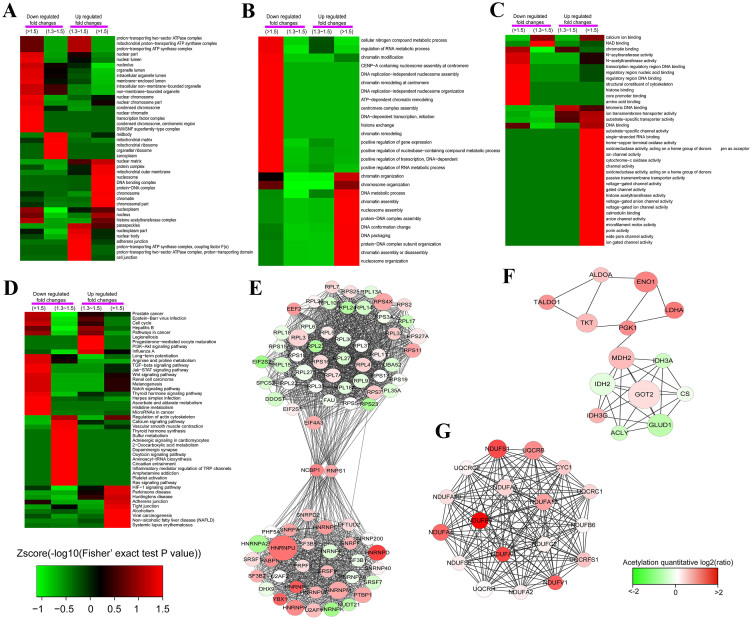
Functional enrichment-based clustering analysis for the quantified acetylome. (A), cellular component analysis. (B), biological process analysis. (C), molecular function analysis. (D), KEGG pathway analysis. (E), protein-protein interaction network of acetylated proteins clustered in ribosome and spliceosome. (F), protein-protein interaction network of acetylated proteins clustered in TCA cycle. (G), protein-protein interaction network of acetylated proteins clustered in oxidation phosphorylation.

The enrichment-based clustering analyses were carried out to compare the functions of corresponding proteins with up- and down-regulated acetylation levels. (Figure 4A–D and Figure S4). It was found that the acetylation level was considerably up-regulated for those proteins involved in histone acetyltransferase complex from GO analysis of cellular component and molecular function (Figure 4A and 4C). However, the acetylation level of some N-acetyltransferases was decreased (Figure 4C). This may be the reason of the existing of down-regulated acetylation sites. For the protein with up-regulated acetylation level, we found that they are associated with transcription such as transcription factor complex in cellular component, DNA-dependent transcription and positive regulation of transcription in biological process, and transcription regulatory region DNA binding in molecular function were all down-regulated (Figure 4A–C). Moreover, proteins related to DNA replication, chromatin remodeling, and gene expression were also quantified with down-regulated acetylation sites (Figure 4B). The KEGG pathway analysis showed that proteins with differentially changed acetylation level were enriched in several important diseases such as Parkinsons disease, Huntingtons disease and prostate cancer (Figure 4C). Apart from above analysis, clustering analysis of protein domain and protein complex for lysine acetylation were all performed as shown in Figure S3A and S3B.

The protein-protein interaction network for acetylome in A549 cells was established by Cytoscape software (Figure S5 and Figure 4E–G). The global scope of network among Kac proteins were first obtained (Figure S5), then Kac proteins were clustered into multiple biological processes (Figure 4E–G). It was observed that Kac proteins participated in ribosome and spliceosome, TCA cycle and oxidation phosphorylation. Some important acetylated proteins were found such as NCBP1 (Nuclear cap-binding protein subunit 1) and RNPS1 (RNA-binding protein with serine-rich domain 1), these two proteins act as connectors between ribosome and spliceosome and were both with up-regulated acetylation level upon SAHA treatment (Figure 4E). Moreover, for TCA cycle, there are 14 enzymes quantified to be acetylated, 9 of which were up-regulated and 5 were down-regulated in acetylation level (Figure 4F). In oxidation phosphorylation, 18 proteins were identified as acetylated protein and all of them were quantified with up-regulated acetylation level upon SAHA treatment (Figure 4G).

### Crosstalk between quantitative ubiquitylome and acetylome

Ubiquitination and acetylation were reported to exist crosstalks^26^. However, no experimental evidence was shown. In this work, we compared the data of acetylome and ubiquitylome obtained from SAHA treated A549 cells to study the crosstalk between acetylation and ubiquitination.

**Figure 5:**
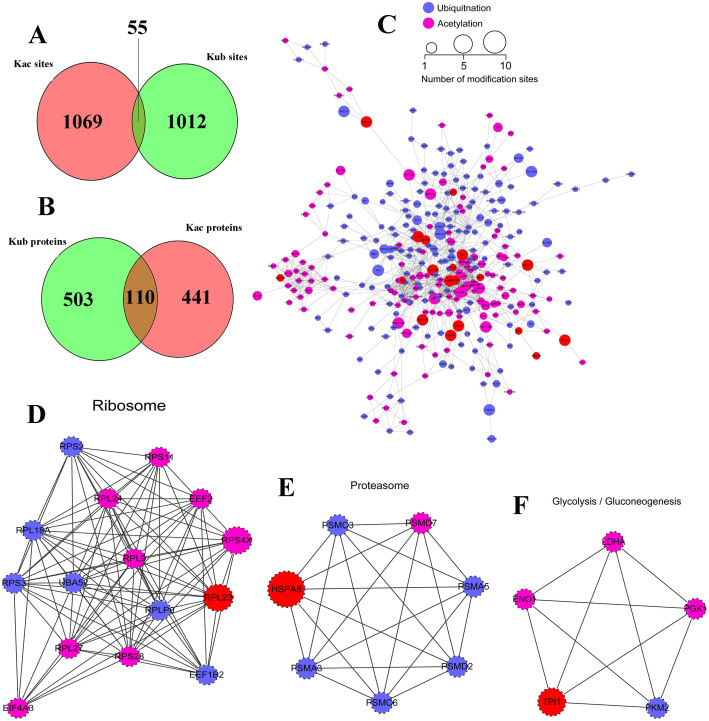
Crosstalk analysis between ubiquitylome and acetylome. (A), overlap between ubiquitination sites and acetylation sites. (B), overlap between ubiquitylated proteins and acetylated proteins. (C), protein-protein interaction network between ubiquitylated proteins and acetylated proteins obtained by Cytoscape. (D), protein-protein interaction network of ubiquitylated and acetylated proteins clustered in ribosome. (E), protein-protein interaction network of ubiquitylated and acetylated proteins clustered in proteasome. (F), protein-protein interaction network of ubiquitylated and acetylated proteins clustered in glycolysis/gluconeogenesis.

**Figure 6:**
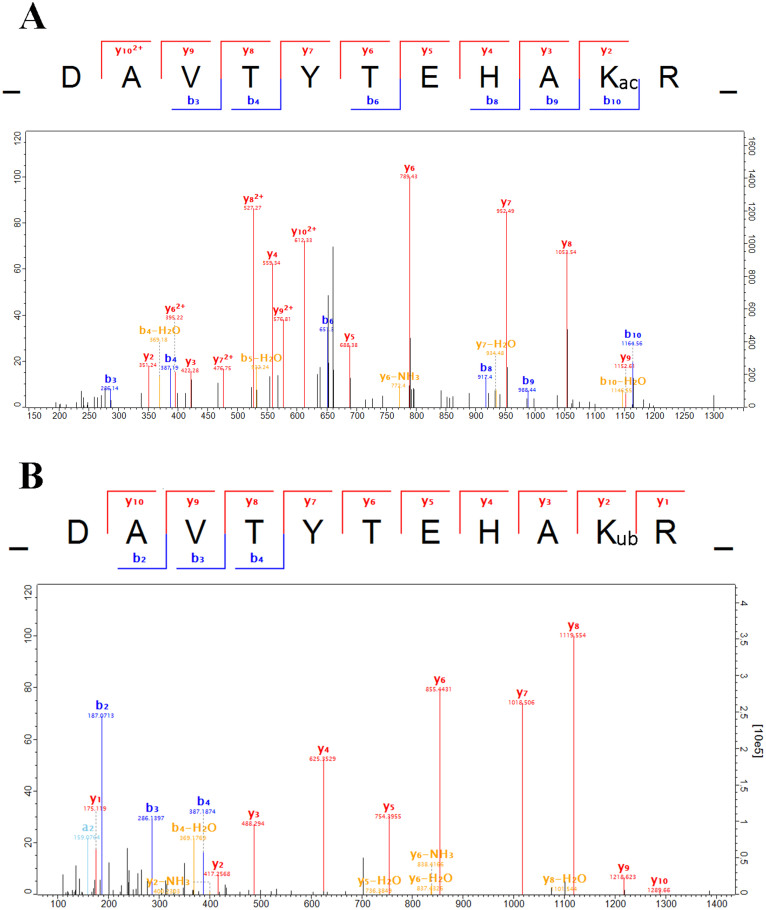
MS/MS spectra of Histone H4K77 acetylation and ubiquitination. (A), acetylation. (B), ubiquitination. This site was both undergo acetylation and ubiquitination.

By comparing the data of Kac and Kub, we identified 55 sites both modified with Kac and Kub, among which 52 sites were quantified. Moreover, there are also 110 proteins that undergo acetylation and ubiquitination at the same protein by at different sites (Figure 5A–B). For the sites both acetylated and ubiquitylated, the changing patterns are always the same (43 out of 52 sites) as shown in Figure 3B, which suggest acetylation and ubiquitination in those proteins are positively related. However, in a few proteins, the changing patterns of Kac and Kub are opposite, such as Histone H3 K122, Nascent polypeptide-associated complex K142 and Acyl-CoA-binding protein K77. Some spectra of lysine sites undergo both acetylation and ubiquitination were presented in Figure 6 and Figure S6.

Many other researchers also studied the crosstalk among various PTM and drawn different conclusion. Pan and Trinidadet al. reported that PTM crosstalks are not significant without any natural selection^36^,^37^ while Grayet al. suggested that PTM crosstalks are significantly under natural selection^38^. The discrepant conclusion of various reports may be related with the different PTM types they studied. Besides, species and even tissues specificity may also affect the PTM crosstalk as different species and tissues were used in their studies. In our study, though the sites both modified with Kac and Kub were not so much, the changing patterns of acetylation and ubiquitination on these sites were positive related (Figure 3B). The crosstalk between acetylome and ubiquitylome in A549 cells under SAHA treatment tend to be positively correlated. However, the precedence rule of acetylation and ubiquitination on these sites demonstrating which modification appears first and how the subsequent modification follows was still unclear and need further investigation.

To deeper reveal the crosstalk between acetylome and ubiquitylome, the protein-protein interaction network based on Kac and Kub proteins were established as shown in Figure 5C–F. The global overview of protein-protein interaction network among Kac and Kub proteins were first obtained by using Cytoscape software (Figure 5C), then Kac and Kub proteins were clustered into multiple biological processes (Figure 5D and 5F). It was observed that Kac and Kub proteins both participated in ribosome, proteasome and glycolysis pathway. Some important proteins involved in these networks are both acetylated and ubiquitylated, for example RPL23 (60S ribosomal protein L23), HSPA8 (heat shock 70 kDa protein) and TPI1 (triosephosphate isomerase). These proteins may of key importance in SAHA treatment of A549 cells and could be selected for further biological investigation.

## Discussion

The expression level of the whole proteome was reported to be changed by SAHA treatment both up-regulated and down-regulated^13^, however, the regulation mechanism of proteome alteration upon SAHA treatment was unexplored. As it is well known that proteins undergo ubiquitination will be degraded by the proteasome due to the biological function of ubiquitination, we proposed previously that SAHA treatment mediated ubiquitination pathway probably is an unrevealed mechanism to regulate the proteome in A549 cells. In this work, the quantitative proteome and ubiquitylome were both obtained upon SAHA treatment in A549 cells. We found that SAHA treatment could largely change protein ubiquitination level both increase and decrease. Finally, by comparing the quantitative results of proteome and ubiquitylome, we revealed that the expression levels of proteins in global proteome are negatively related to the ubiquitination levels in the same proteins (Figure 3A). That means when ubiquitination level of a specific protein increases, the level of this protein correspondingly decreases. It is completely consistent with the protein-degradation function of ubiquitination. Therefore, we came to a conclusion that the alteration of proteome expression level upon SAHA treatment is regulated or partially regulated by protein ubiquitination.

In this work, the quantitative acetylome of A549 cells before and after SAHA treatment was also obtained with more than 1000 Kac sites being quantified. It is the most comprehensive Kac profiling upon SAHA treatment in A549 cells by now. Moreover, 91 Kac sites were identified in histones, which is also the largest dataset of histone acetylation upon SAHA treatment. Due to the treatment of SAHA, the acetylation levels of almost all histones were greatly increased with the largest quantitative ratio of 44.26 in histone H2B K5. However, there were still three histone Kac sites quantified as down-regulated acetylation level, including H3 K23, H4 K12 and H4 K79, two of which were already reported previously^13^. For this interesting phenomenon, further study should be carried out to reveal the hiding principle. For non-histone proteins, the acetylation was also largely increased after SAHA treatment, but not so extensively increased as in histones. There are still quite a number of proteins with down-regulated acetylation level. As SAHA is the inhibitor of histone deacetylases, its activity to non-histone proteins may be compromised. We found that the acetylation levels of N-acetyltransferases such as N-acetyltransferase 10, N-alpha-acetyltransferase 30 and CREB-binding protein are down-regulated by SAHA treatment. This could be the reason for the decrease of acetylation level in non-histone proteins. For example, CREB-binding protein acetylates both histone and non-histone proteins, the decrease of acetylation level in histone H3 K23, H4 K12 and H4 K79 may regulated by this enzyme.

Lastly, the crosstalk between acetylome and ubiquitylome was also analyzed. According to our results, for the sites both acetylated and ubiquitylated, Kac was positively related to Kub in most sites (43 out of 52 sites) as shown in Figure 3B. As SAHA treatment could largely increase the acetylation level in cells, the ubiquitination level will also be increased due to the crosstalk. Then, the expression level of the proteins being ubiquitylated is decreased by protein degradation. As a result, the up-regulation of acetylation level by SAHA treatment in cells eventually induced the down-regulation of global proteome expression level which was also reported previously^26^. Finally, we conclude the interaction of acetylome, ubiquitination and global proteome with positive regulation between acetylome and ubiquitylome, and negative regulation between ubiquitylome and global proteome.

**Figure 7:**
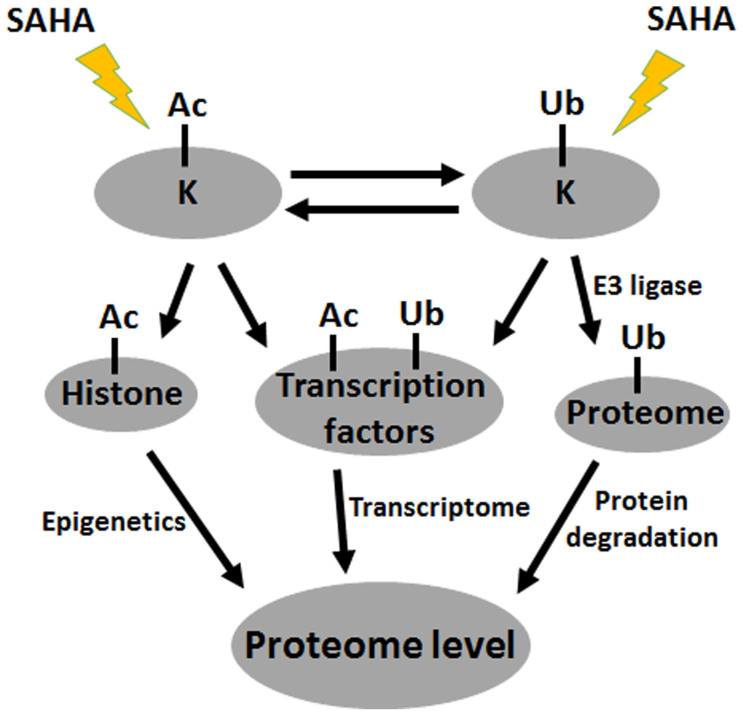
Schematic diagram of the relationship among global proteome, ubiquitylome and acetylome.

According to the data of this study, the relationship among global proteome, ubiquitylome and acetylome were summarized in Figure 7. Firstly, SAHA treatment both directly changes lysine acetylation and ubiquitination level in A549 cells. Moreover, the alteration level of acetylation and ubiquitination will also regulate each other due to the existed crosstalks between acetylation and ubiquitination. Secondly, the changing level of protein lysine acetylation could induce the change of global proteome level in two aspects. Lysine acetylation may divide into two types, histone acetylation and non-histone acetylation. Histone acetylation could induce the change of proteome level by epigenetics as previously reported^13^, while non-histone acetylation of transcription factors could regulated proteome level through transcriptome. Thirdly, protein lysine ubiquitination could also induce the change of global proteome level. The ubiquitination level of proteins could be induced by E3 ligase and then degraded by proteasome. Moreover, transcription factors could also undergo ubiquitination and induce the global proteome level through transcriptome. Above is just the predicted mechanism, for further confirmation, more experimental results should be obtained.

In conclusion, we comprehensively investigated the effects of SAHA on A549 cells. Taking the advantages of SILAC labeling, antibody-based affinity enrichment and high-resolution LC-MS/MS, quantitative comparison of the proteome, ubiquitylome and acetylome before and after SAHA treatment were extensively studied. It was found that SAHA broadly changed the proteome, ubiquitylome and acetylome of A549 cells. By the help of advanced bioinformatic analysis, important biological processes and functions related with SAHA were revealed. More importantly, the crosstalk among global proteome, ubiquitylome and acetylome were also studied, which may considerably expand our current understanding of SAHA-dependent NSCLC therapy.

## Methods

### Stable isotope labeling and SAHA treatment in A549 cells

Non-small cell lung cancer (NSCLC) cell line A549 cells (American Type Culture Collection, ATCC; Catalog CCL-185; Manassas, VA) were maintained in DMEM SILAC medium (Invitrogen, Carlsbad, CA) supplemented with 10% FBS (Life Technologies, Grand Island, NY) at 37°C in humidified atmosphere with 5% CO_2_.

The stable isotope labeling was performed as described previously^13^. In brief, A549 cells were maintained in SILAC Protein Quantitation Kit (Invitrogen, Carlsbad, CA) according to manufacturer's instructions. Briefly, cells were maintained in DMEM culture medium supplemented with 10% fetal bovine serum (FBS) supplemented with either the “heavy (H)” form of [U−13C6]-L-lysine or “light (L)” [U−12C6]-L-lysine for over six generations. The heavy labeling efficiency was evaluated by mass spectrometer analysis to a confirmed >97% labeling efficiency. After that, the cells were further expanded in SILAC media to desired cell number (~5 × 10^8^) in twenty 150 cm^2^ flasks. The “light” labeled cells were then treated with SAHA at 3 μM final concentrations for 18 hours and the “heavy” labeled cells treated with same volume of DMSO for 18 hours, respectively. The SAHA concentration and duration time were determined according to our previous report^13^. After treatment, the cells were harvested and washed twice with ice-cold PBS supplemented with 2 μM Trichostatin A and 30 mM Nicotinamide. After snap freezing in liquid nitrogen, cell pellets were stored in −80°C freezer. Extraction of proteins was followed the method described previously^13^.

### In-solution digestion and HPLC fractionation

For reduction of proteins, dithothreitol (DTT) was then added to final concentration 10 mM followed by incubation at 56°C for 60 min. After that, iodoacetamine (IAA) was added to alkylate proteins to final concentration 15 mM followed by incubation at room temperature in dark for 40 min. The alkylation reaction was quenched by 30 mM of cysteine (final concentration) at room temperature for another 30 min. Trypsin was then added with the ratio of trypsin to protein at 1:25 (w/w) for digestion at 37°C for overnight.

The protein digestion was then fractionated by high pH reverse-phase HPLC using Agilent 300Extend C18 column (5 μm particles, 4.6 mm ID, 250 mm length). Briefly, peptides were first separated with a gradient of 2% to 60% acetonitrile in 10 mM ammonium bicarbonate pH 8 over 80 min into 80 fractions. Then, the peptides were combined into 18 fractions for the global proteome analysis as previously reported^39^. For ubiquitination and acetylation analysis, no HPLC fractionation was performed.

### Affinity enrichment of lysine acetylated and ubiquitylated peptides

Prior to affinity enrichment, anti-lysine acetylation (Kac) and anti-lysine ubiquitination (Kub) antibody beads (PTM Biolabs, Inc, Hangzhou) were washed twice with ice-cold PBS. To enrich Kac and Kub peptides, 5 mg tryptic peptides of Kac and Kub were dissolved in NETN buffer (100 mM NaCl, 1 mM EDTA, 50 mM Tris-HCl, 0.5% NP-40, pH 8.0) and then incubated separately with pre-washed antibody beads (catalog no. PTM-104 for Kac and catalog no. PTM-1104 for Kub, PTM Biolabs, Inc, Hangzhou, respectively) in a ratio of 15 μL beads/mg protein at 4°C overnight with gentle shaking. The beads were washed four times with NETN buffer and twice with ddH_2_O. The bound peptides were eluted from the beads with 0.1% TFA. The eluted peptides were collected and vacuum-dried followed by LC-MS/MS analysis.

### LC-MS/MS analysis

Peptides were re-dissolved in solvent A (0.1% FA in 2% ACN) and directly loaded onto a reversed-phase pre-column (Acclaim PepMap 100, Thermo Scientific). Peptide separation was performed using a reversed-phase analytical column (Acclaim PepMap RSLC, Thermo Scientific) with a linear gradient of 5–35% solvent B (0.1% FA in 98% ACN) for 30 min and 35–80% solvent B for 10 min at a constant flow rate of 300 nl/min on an EASY-nLC 1000 UPLC system. The resulting peptides were analyzed by Q Exactive™ Plus hybrid quadrupole-Orbitrap mass spectrometer (Thermo Fisher Scientific).

The peptides were subjected to NSI source followed by tandem mass spectrometry (MS/MS) in Q Exactive™ Plus (Thermo) coupled online to the UPLC. Intact peptides were detected in the Orbitrap at a resolution of 70,000. Peptides were selected for MS/MS using 28% NCE; ion fragments were detected in the Orbitrap at a resolution of 17,500. A data-dependent procedure that alternated between one MS scan followed by 20 MS/MS scans was applied for the top 20 precursor ions above a threshold ion count of 2E4 in the MS survey scan with 15.0 s dynamic exclusion. The electrospray voltage applied was 2.0 kV. Automatic gain control (AGC) was used to prevent overfilling of the ion trap; 5E4 ions were accumulated for generation of MS/MS spectra. For MS scans, the m/z scan range was 350 to 1600.

### Database search

The protein, acetylation site and ubiquitination sites identification and quantification were performed through MaxQuant with an integrated Andromeda search engine (version 1.4.1.2). Tandem mass spectra were searched against SwissProt Human database (20,274 sequences) concatenated with reverse decoy database and protein sequences of common contaminants. Trypsin/P was specified as cleavage enzyme allowing up to 3 missing cleavages, 4 modifications per peptide and 5 charges. Mass error was set to 6 ppm for precursor ions and 0.02 Da for fragment ions. Carbamidomethylation on Cys was specified as fixed modification and oxidation on Met, acetylation and ubiquitination on lysine and acetylation on protein N-terminal were specified as variable modifications. False discovery rate (FDR) thresholds for protein, peptide and modification site were specified at 0.01. Minimum peptide length was set at 7. All the other parameters in MaxQuant were set to default values. Kac and Kub site identifications with localization probability less than 0.75 or from reverse and contaminant protein sequences were removed.

For quantification of the SILAC data (Heavy/Light ratio calculation), the built-in SILAC 2-plex quantification method was used (Proteome Discoverer 1.3, Thermo Fisher) withLys-0 and Lys-6 labels, based on ion intensities of monoisotopic peaks observed in the LC MS spectra. To minimize the systematic errors introduced by the Bradford assay and sample mixing, the normalization was done using a multiple-point normalization strategy according to previous report^40^. Briefly, the distributions of protein ratios were plotted by SPSS statistical software (version 12.0, IBM Company, Chicago, IL, USA), then 5% trimmed mean values were calculated. All protein ratios were normalized against the 5% trimmed mean values so that most protein ratios were distributed in the 1.00 ± 0.10 zone. Data for SAHA treated and untreated cells labeled with ‘light’ or ‘heavy’ amino acids were combined for identifying significant changes in level of proteome, acetylome and ubiquitylome. Comparisons between variables were tested by paired t test and p values < 0.05 were considered to be statistically significant.

### Bioinformatic analysis

Gene Ontology (GO) term association and enrichment analysis were performed using the Database for Annotation, Visualization and Integrated Discovery (DAVID). Encyclopedia of Genes and Genomes (KEGG) database was used to identify enriched pathways by Functional Annotation Tool of DAVID against the background of Homo sapiens. InterPro database was researched using Functional Annotation Tool of DAVID against the background of Homo sapiens. Manually curated CORUM protein complex database for human was used for protein complex analysis. To construct a protein–protein interaction network, the STRING database system was used. Functional protein interaction networks were visualized using Cytoscape. When performing the bioinformatics analysis, corrected p-value < 0.05 was considered significant. And all the detailed description of bioinformatic analysis was listed in Supplementary Information.

## Author Contributions

Q.W., P.S.F. and Z.Y.C. designed experiments; J.Z., W.Q.X., C.B.C., W.T.L. and X.L.Y. performed the experiments; L.J.C., F.S.W., Z.W.W. and J.L. helped to perform the experiments; X.J.P. analyzed data; Q.W., Z.Y.C. and J.Z. wrote the manuscript. All authors reviewed the manuscript.

## Supplementary Material

Supplementary Information Supplementary information

Supplementary Information Supplementary Table S1

Supplementary Information Supplementary Table S2

Supplementary Information Supplementary Table S3

Supplementary Information Supplementary Table S4
