# Supplementary material for: Suberoylanilide Hydroxamic Acid Treatment Reveals Crosstalks among Proteome, Ubiquitylome and Acetylome in Non-Small Cell Lung Cancer A549 Cell Line
Source: Sci Rep. 2015 Mar 31;5:9520. doi: 10.1038/srep09520 (PMC4379480; doi:10.1038/srep09520)
Supplement: Supplementary Information [file srep09520-s1.pdf]

Suberoylanilide Hydroxamic Acid Treatment Reveals Crosstalks among Proteome, Ubiquitylome and Acetylome in Non-Small Cell Lung Cancer A549 Cell Line

Quan Wu[1], Zhongyi Cheng[2], Jun Zhu[3], Weiqing Xu[1], Xiaojun Peng[3], Chuangbin Chen[3], Wenting Li[1], Fengsong Wang[4], Lejie Cao[5], Xingling Yi[3], Zhiwei Wu[1], Jing Li[1], Pingsheng Fan[6]

[1] Central Laboratory of Medical Research Centre, Affiliated Provincial Hospital, Anhui Medical University, Hefei, 230001, China

[2] Institute for Advanced Study of Translational Medicine, Tongji University, Shanghai, 200092, China

[3] Jingjie PTM Biolab (Hangzhou) Co. Ltd, Hangzhou 310018, China

[4] School of Life science, Anhui Medical University, Hefei, 230032, China

[5] Department of Respiration, Affiliated Provincial Hospital, Anhui Medical University, Hefei, 230001, China

[6] Department of Oncology, Affiliated Provincial Hospital, Anhui Medical University, Hefei, 230001, China

Correspondence and requests for materials should be addressed to

Q.W. (powerwoo01@ahmu.edu.cn, Ph: +86-551-62283574, fax: +86-551-62283292) or P.S.F. (fanpingsheng@csco.org.cn, Ph: +86-551-65897876, fax: +86-551-62283292)

Bioinformatic Analysis

Enrichment of Gene Ontology analysis. Proteins were classified by GO annotation into three categories: biological process, cellular compartment and molecular function. For each category, we used Functional Annotation Tool of DAVID Bioinformatics Resources 6.7 to identify enriched GO against the background of Homo sapiens. A two-tailed Fisher's exact test was employed to test the enrichment of the protein-containing IPI entries against all IPI proteins. Correction for multiple hypothesis testing was carried out using standard false discovery rate control methods. The GO with a corrected p-value < 0.05 is considered significant.

Enrichment of pathway analysis. Encyclopedia of Genes and Genomes (KEGG) database was used to identify enriched pathways by Functional Annotation Tool of DAVID against the background of Homo sapiens. A two-tailed Fisher's exact test was employed to test the enrichment of the protein-containing IPI entries against all IPI proteins. Correction for multiple hypothesis testing was carried out using standard false discovery rate control methods. The pathway with a corrected p-value $<$ 0.05 was considered significant. These pathways were classified into hierarchical categories according to the KEGG website.

Enrichment of protein domain analysis. For each category proteins, InterPro (a resource that provides functional analysis of protein sequences by classifying them into families and predicting the presence of domains and important sites) database was researched using Functional Annotation Tool of DAVID against the background of Homo sapiens. A two-tailed Fisher's exact test was employed to

test the enrichment of the protein-containing IPI entries against all IPI proteins. Correction for multiple hypothesis testing was carried out using standard false discovery rate control methods and domains with a corrected p-value < 0.05 were considered significant.

Enrichment of complex analysis. Manually curated CORUM protein complex database for human was used for protein complex analysis. Overrepresented complexes were identified using hypergeometric test for each category proteins. A two-tailed Fisher's exact test was employed to test the enrichment of the protein-containing SwissProt entries against all SwissPort human proteins. Correction for multiple hypothesis testing was carried out using standard false discovery rate control methods and complexes with a corrected p-value < 0.05 were considered significant.

Enrichment-based clustering analysis. All the protein categories obtained after enrichment were collated along with their P values, and then filtered for those categories which were at least enriched in one of the clusters with P value<0.05. This filtered P value matrix was transformed by the function $x = -\log10$ (P value). Finally these x values were z-transformed for each category. These z scores were then clustered by one-way hierarchical clustering (Euclidean distance, average linkage clustering) in Genesis. Cluster membership was visualized by a heat map using the "heatmap.2" function from the "gplots" R-package.

Protein-protein interaction network analysis. We analyzed protein-protein interaction for identified proteins using Cytoscape software.

Protein-protein interaction network obtained from STRING database. STRING defines a metric called “confidence score” to define interaction confidence; we fetched all interactions that had a confidence score ≥ 0.7 (high confidence). Interaction network form STRING was visualized in Cytoscape. A graph the oretical clustering algorithm, molecular complex detection (MCODE) was utilized to analyzed densely connected regions. MCODE is part of the plug-in tool kit of the network analysis and visualization software Cytoscape.

Figure Legends

Figure S1. Functional enrichment-based clustering analysis for the quantified proteome. A, cellular component analysis. B, biological process analysis. C, molecular function analysis. D, protein domain analysis. E, KEGG pathway analysis. F, protein complex analysis.

Figure S2. Functional enrichment-based clustering analysis for the quantified ubiquitylome. A, protein domain analysis. B, protein complex analysis.

Figure S3. Protein-protein interaction network of ubiquitylome established using Cytoscape.

Figure S4. Functional enrichment-based clustering analysis for the quantified acetylome. A, protein domain analysis. B, protein complex analysis.

Figure S5. Protein-protein interaction network of acetylome established using Cytoscape.

Figure S6. MS/MS spectra of HSPA8 K601 acetylation and ubiquitination. A, acetylation. B, ubiquitination. This site was both undergo acetylation and ubiquitination.

Figure S1

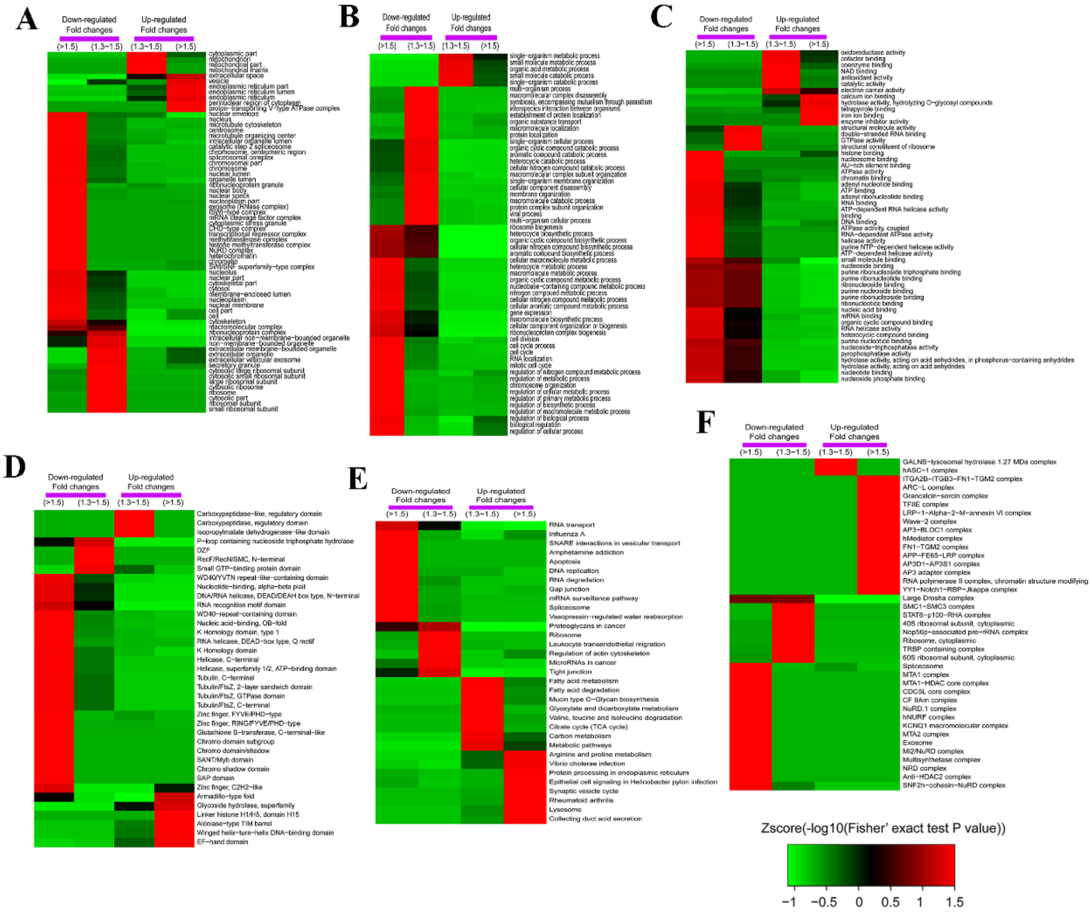
A
B
C
D
E
F
Down-regulated
Fold changes
Up-regulated
Fold changes
(>1.5)
(1.3~1.5)
(1.3~1.5)
(>1.5)
Zscore(-log10(Fisher' exact test P value))
−1
−0.5
0
0.5
1
1.5

Figure S2

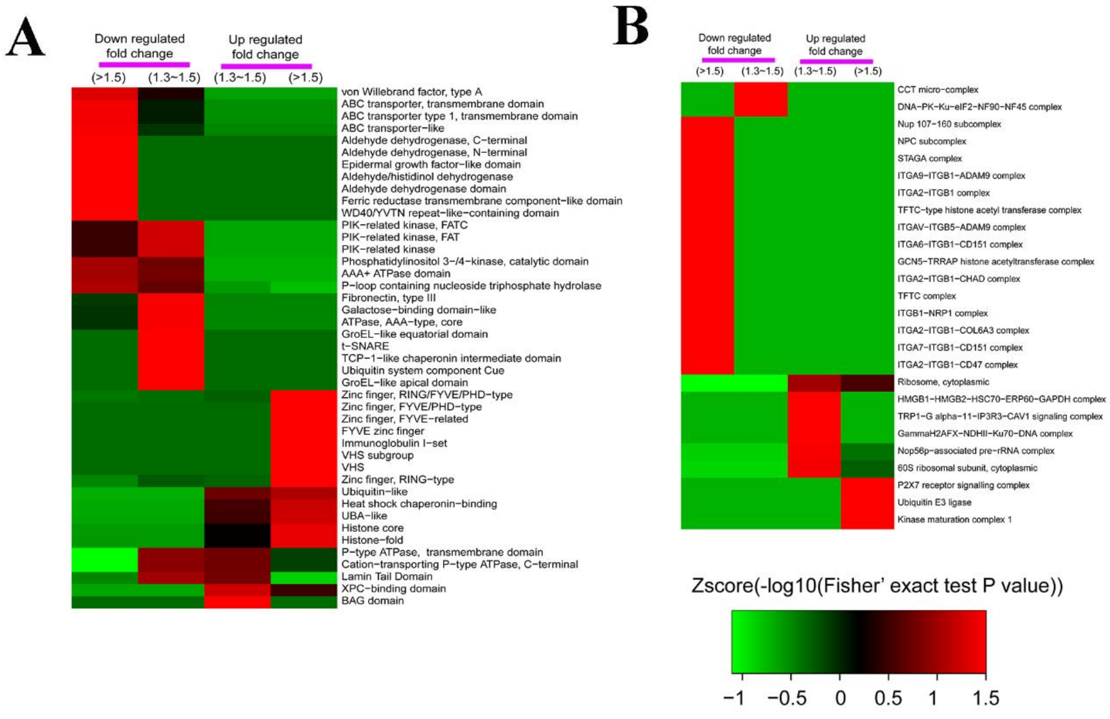

A
Down regulated fold change
Up regulated fold change
(>1.5)
(1.3~1.5)
(1.3~1.5)
(>1.5)
von Willebrand factor, type A
ABC transporter, transmembrane domain
ABC transporter type 1, transmembrane domain
ABC transporter-like
Aldehyde dehydrogenase, C-terminal
Aldehyde dehydrogenase, N-terminal
Epidermal growth factor-like domain
Aldehyde/histidinol dehydrogenase
Aldehyde dehydrogenase domain
Ferric reductase transmembrane component-like domain
WD40/YVTN repeat-like-containing domain
PIK-related kinase, FATC
PIK-related kinase, FAT
PIK-related kinase
Phosphatidylinositol 3-/4-kinase, catalytic domain
AAA+ ATPase domain
P-loop containing nucleoside triphosphate hydrolase
Fibronectin, type III
Galactose-binding domain-like
ATPase, AAA-type, core
GroEL-like equatorial domain
t-SNARE
TCP-1-like chaperonin intermediate domain
Ubiquitin system component Cue
GroEL-like apical domain
Zinc finger, RING/FYVE/PHD-type
Zinc finger, FYVE/PHD-type
Zinc finger, FYVE-related
FYVE zinc finger
Immunoglobulin I-set
VHS subgroup
VHS
Zinc finger, RING-type
Ubiquitin-like
Heat shock chaperonin-binding
UBA-like
Histone core
Histone-fold
P-type ATPase, transmembrane domain
Cation-transporting P-type ATPase, C-terminal
Lamin Tail Domain
XPC-binding domain
BAG domain
B
Down regulated fold change
Up regulated fold change
(>1.5)
(1.3~1.5)
(1.3~1.5)
(>1.5)
CCT micro-complex
DNA-PK-Ku-eIF2-NF90-NF45 complex
Nup 107-160 subcomplex
NPC subcomplex
STAGA complex
ITGA9-ITGB1-ADAM9 complex
ITGA2-ITGB1 complex
TFTC-type histone acetyl transferase complex
ITGAV-ITGB5-ADAM9 complex
ITGA6-ITGB1-CD151 complex
GCN5-TRRAP histone acetyltransferase complex
ITGA2-ITGB1-CHAD complex
TFTC complex
ITGB1-NRP1 complex
ITGA2-ITGB1-COL6A3 complex
ITGA7-ITGB1-CD151 complex
ITGA2-ITGB1-CD47 complex
Ribosome, cytoplasmic
HMGB1-HMGB2-HSC70-ERP60-GAPDH complex
TRP1-G alpha-11-IP3R3-CAV1 signaling complex
GammaH2AFX-NDHII-Ku70-DNA complex
Nop56p-associated pre-rRNA complex
60S ribosomal subunit, cytoplasmic
P2X7 receptor signalling complex
Ubiquitin E3 ligase
Kinase maturation complex 1
Zscore(-log10(Fisher' exact test P value))
-1
-0.5
0
0.5
1
1.5

Figure S3

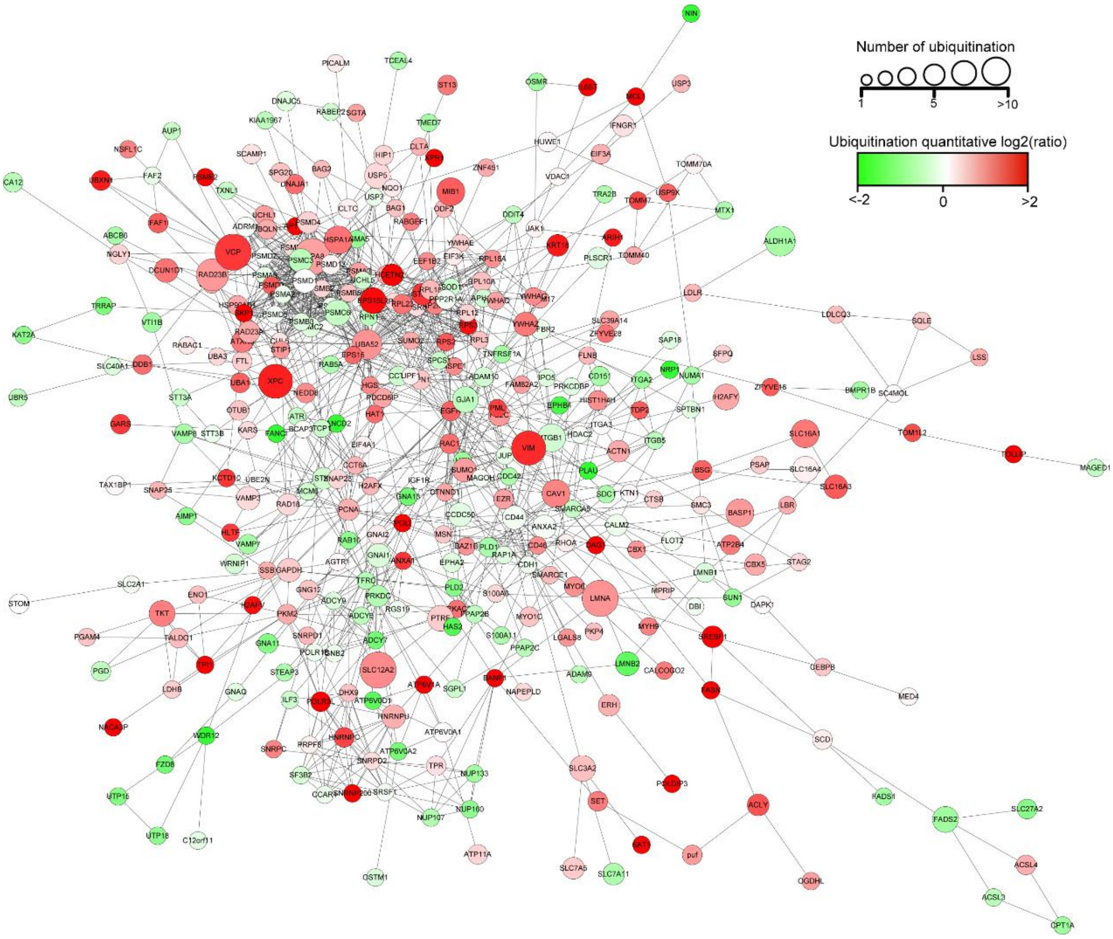
Number of ubiquitination
1
5
>10
Ubiquitination quantitative log2(ratio)
<-2
0
>2

Figure S4

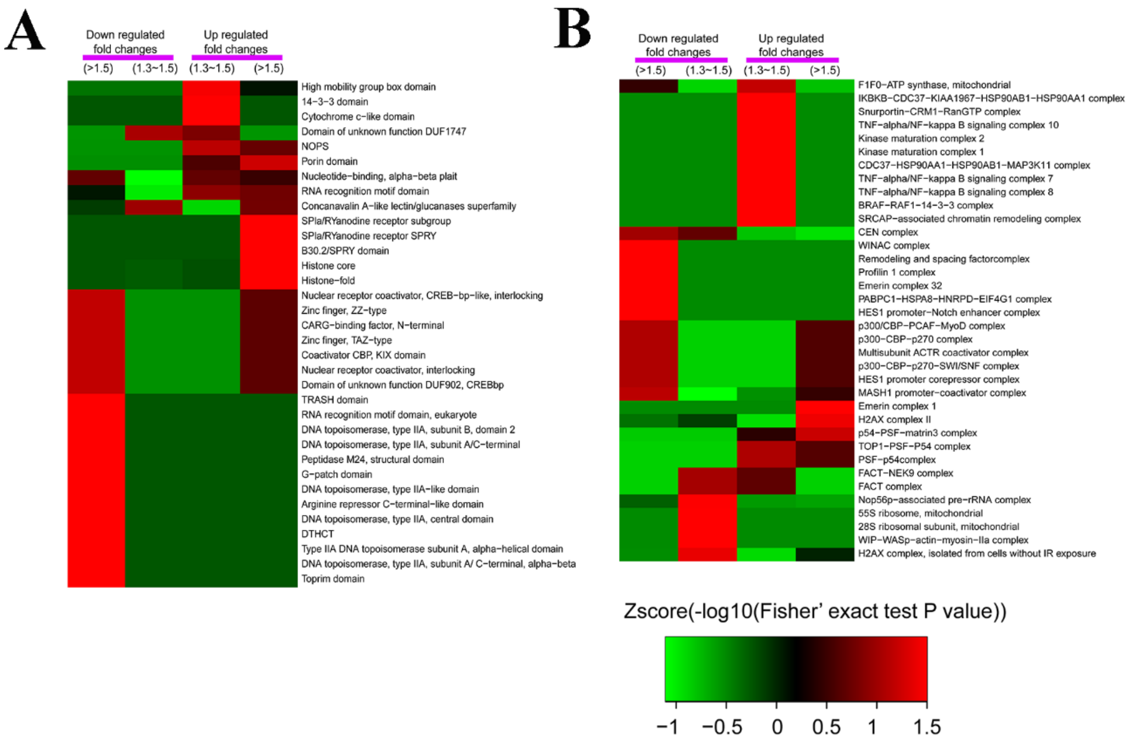
A
Down regulated fold changes
Up regulated fold changes
(>1.5)
(1.3~1.5)
(1.3~1.5)
(>1.5)
High mobility group box domain
14−3−3 domain
Cytochrome c−like domain
Domain of unknown function DUF1747
NOPS
Porin domain
Nucleotide−binding, alpha−beta plait
RNA recognition motif domain
Concanavalin A−like lectin/glucanases superfamily
SPla/RYanodine receptor subgroup
SPla/RYanodine receptor SPRY
B30.2/SPRY domain
Histone core
Histone−fold
Nuclear receptor coactivator, CREB−bp−like, interlocking
Zinc finger, ZZ−type
CARG−binding factor, N−terminal
Zinc finger, TAZ−type
Coactivator CBP, KIX domain
Nuclear receptor coactivator, interlocking
Domain of unknown function DUF902, CREBbp
TRASH domain
RNA recognition motif domain, eukaryote
DNA topoisomerase, type IIA, subunit B, domain 2
DNA topoisomerase, type IIA, subunit A/C−terminal
Peptidase M24, structural domain
G−patch domain
DNA topoisomerase, type IIA−like domain
Arginine repressor C−terminal−like domain
DNA topoisomerase, type IIA, central domain
DTHCT
Type IIA DNA topoisomerase subunit A, alpha−helical domain
DNA topoisomerase, type IIA, subunit A/ C−terminal, alpha−beta
Toprim domain
B
Down regulated fold changes
Up regulated fold changes
(>1.5)
(1.3~1.5)
(1.3~1.5)
(>1.5)
F1F0−ATP synthase, mitochondrial
IKBKB−CDC37−KIAA1967−HSP90AB1−HSP90AA1 complex
Snurportin−CRM1−RanGTP complex
TNF−alpha/NF−kappa B signaling complex 10
Kinase maturation complex 2
Kinase maturation complex 1
CDC37−HSP90AA1−HSP90AB1−MAP3K11 complex
TNF−alpha/NF−kappa B signaling complex 7
TNF−alpha/NF−kappa B signaling complex 8
BRAF−RAF1−14−3−3 complex
SRCAP−associated chromatin remodeling complex
CEN complex
WINAC complex
Remodeling and spacing factorcomplex
Profilin 1 complex
Emerin complex 32
PABPC1−HSPA8−HNRPD−EIF4G1 complex
HES1 promoter−Notch enhancer complex
p300/CBP−PCAF−MyoD complex
p300−CBP−p270 complex
Multisubunit ACTR coactivator complex
p300−CBP−p270−SWI/SNF complex
HES1 promoter corepressor complex
MASH1 promoter−coactivator complex
Emerin complex 1
H2AX complex II
p54−PSF−matrin3 complex
TOP1−PSF−P54 complex
PSF−p54complex
FACT−NEK9 complex
FACT complex
Nop56p−associated pre−rRNA complex
55S ribosome, mitochondrial
28S ribosomal subunit, mitochondrial
WIP−WASp−actin−myosin−IIa complex
H2AX complex, isolated from cells without IR exposure
Zscore(−log10(Fisher' exact test P value))
−1
−0.5
0
0.5
1
1.5

Figure S5

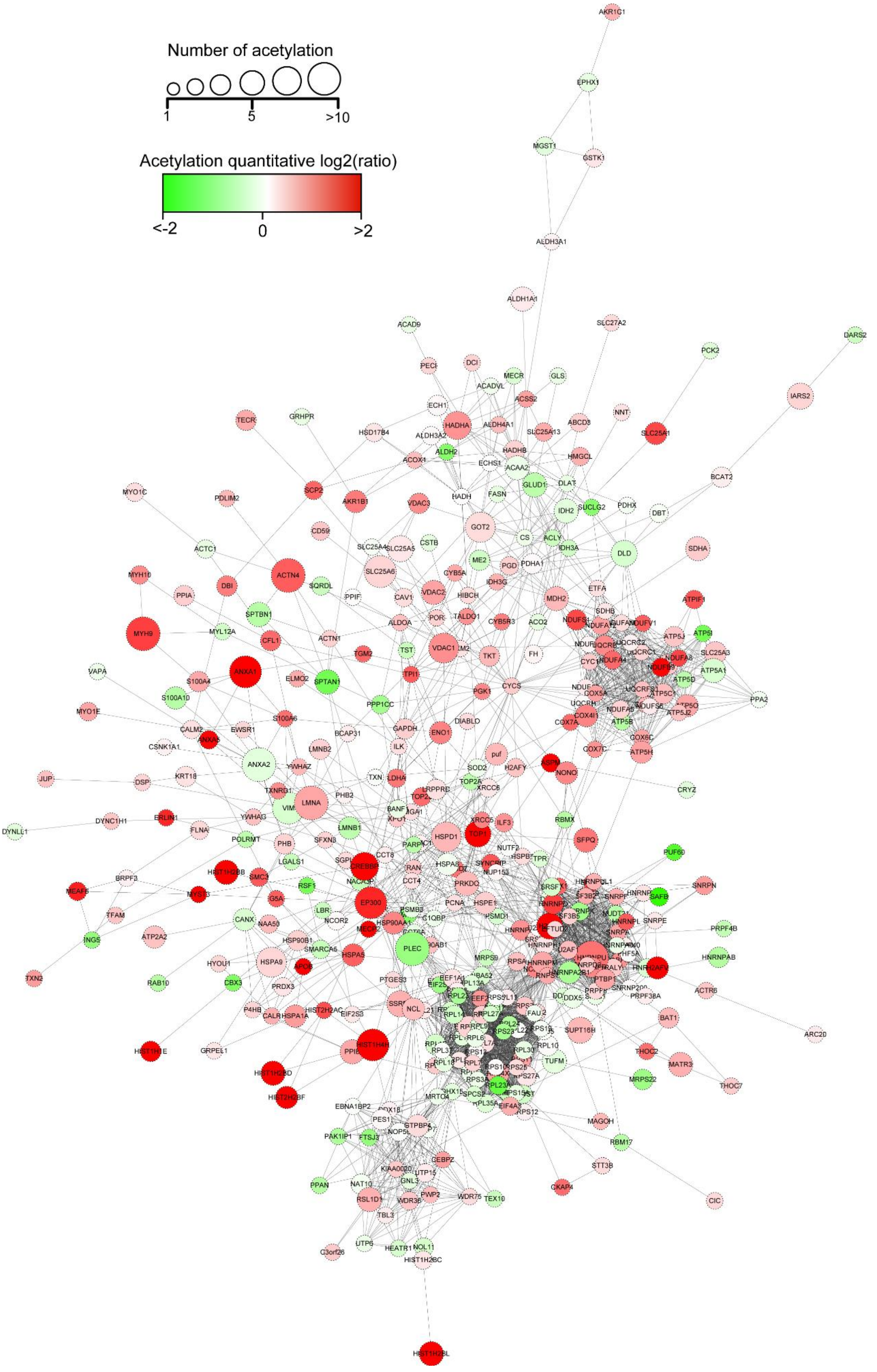
Number of acetylation
1
5
>10
Acetylation quantitative log2(ratio)
<-2
0
>2
AKR1C1
EPHX1
MGST1
GSTK1
ALDH3A1
ALDH1A1
ACAD9
SLC27A2
DARS2
PCK2
IARS2
SLC25A1
BCAT2
TECR
GRHPR
HADHA
ALDH4A1
SLC25A13
ABCD3
HMGCL
ALDH2
ACOX1
SCP2
AKR1B1
VDAC3
GOT2
DLD
SDHA
MYO1C
PDLIM2
ACTN4
MYH10
MYH9
ANXA1
SPTBN1
SPTAN1
CFL1
VDAC1
VDAC2
ATPIF1
ATP5A1
PPA2
VAPA
S100A4
S100A10
MYO1E
ANXA2
LMNA
VIM
JUP
DSP
KRT18
DYNLL1
DYNC1H1
ERLIN1
FLNA
POLRMT
EP300
CREBBP
HSPD1
TOP1
PLEC
HSPA9
HSPA5
NCL
SFPQ
NONO
MEAF6
ING5
TXN2
RAB10
CBX3
HIST1H1E
HIST1H2BD
HIST2H2BF
HIST1H4H
HIST1H2BL
HIST1H2BC
SUPT16H
TUFM
MRPS22
THOC2
THOC7
MATR3
ARC20
BAT1
ACTR6
HNRNPAB
PRPF4B
SNRPN
PUF60
CRYZ
RBM17
STT3B
GKAP1
CIC
MAGOH
PPAN
NAT10
RSL1D1
WDR36
PWP2
WDR75
TEX10
TBL3
GNL3
UTP15
UTP6
HEATR1
NOL11
C3orf26
EBNA1BP2
PES1
PAK1IP1
FTSJ3
GEBPZ

Figure S6

A

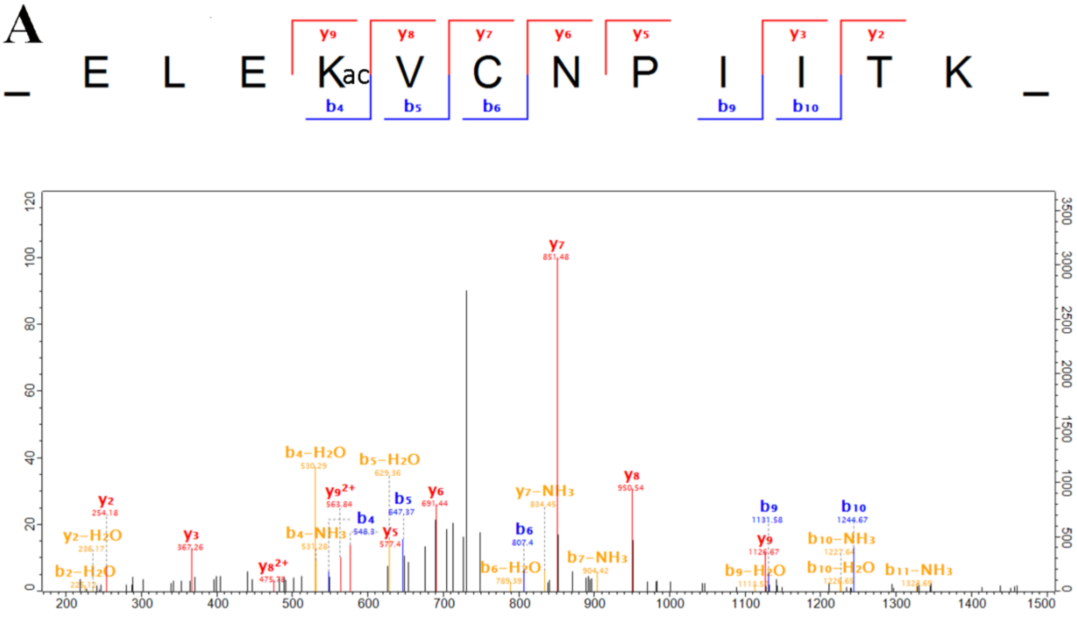
_ E L E Kac V C N P I I T K _
y9 y8 y7 y6 y5 y3 y2
b4 b5 b6 b9 b10
y7
851.48
y8
950.54
b4-H2O
530.29
b5-H2O
629.36
y9 2+
563.84
b5
647.37
y6
691.44
b4
548.3
y5
577.4
b4-NH3
531.28
y7-NH3
834.45
b6
807.4
b7-NH3
904.42
b6-H2O
789.39
y2
254.18
y2-H2O
236.17
y3
367.26
b2-H2O
y8 2+
475.33
b9
1131.58
b10
1244.67
y9
1126.67
b10-NH3
1227.64
b9-H2O
b10-H2O
b11-NH3

B

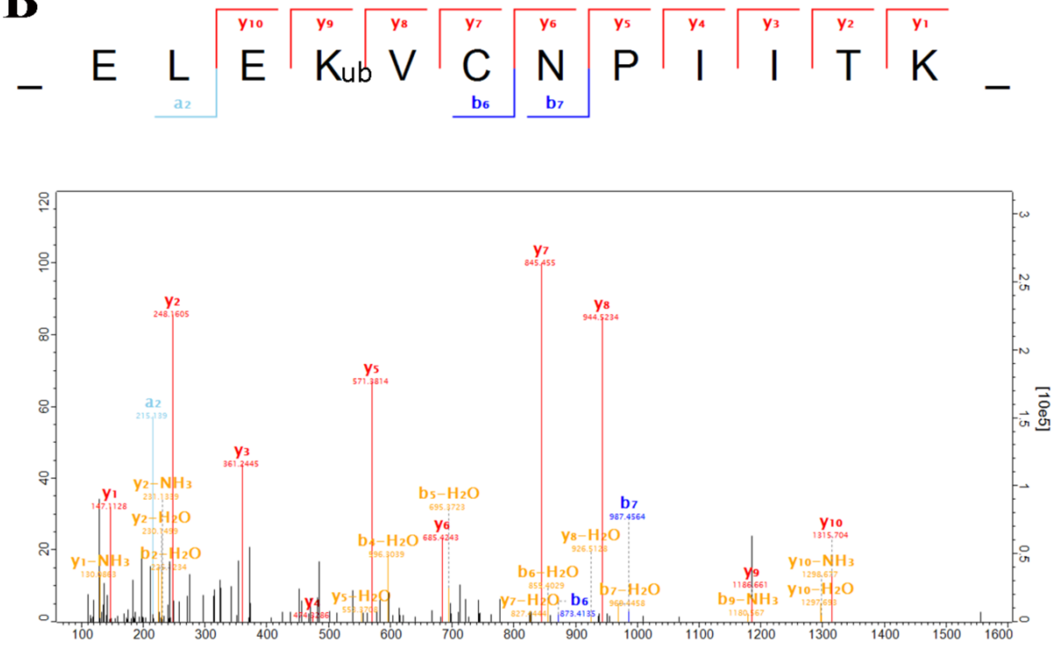
_ E L E Kub V C N P I I T K _
y10 y9 y8 y7 y6 y5 y4 y3 y2 y1
a2 b6 b7
[10e5]
y7
845.455
y8
944.5234
y2
248.1605
y5
571.3814
a2
215.139
y3
361.2445
y1
147.1128
y2-NH3
231.1339
y2-H2O
230.1499
y1-NH3
130.0863
b2-H2O
b5-H2O
695.3723
b4-H2O
596.3039
y6
685.4243
y5-H2O
y4
b7
987.4564
y8-H2O
926.5128
b6-H2O
855.4029
y7-H2O
827.4448
b6
873.4135
b7-H2O
969.4458
y10
1315.704
y9
1186.661
b9-NH3
1180.567
y10-NH3
1298.677
y10-H2O
1297.693
